# Integration of neuromorphic AI in event-driven distributed digitized systems: Concepts and research directions

**DOI:** 10.3389/fnins.2023.1074439

**Published:** 2023-02-17

**Authors:** Mattias Nilsson, Olov Schelén, Anders Lindgren, Ulf Bodin, Cristina Paniagua, Jerker Delsing, Fredrik Sandin

**Affiliations:** ^1^Embedded Intelligent Systems Lab (EISLAB), Department of Computer Science, Electrical and Space Engineering, Luleå University of Technology, Lulea, Sweden; ^2^Applied AI and IoT, Industrial Systems, Digital Systems, RISE Research Institutes of Sweden, Kista, Sweden

**Keywords:** neuromorphic computing, edge intelligence, event-driven systems, non-von Neumann, system integration, microservices, extreme heterogeneity, interoperability

## Abstract

Increasing complexity and data-generation rates in cyber-physical systems and the industrial Internet of things are calling for a corresponding increase in AI capabilities at the resource-constrained edges of the Internet. Meanwhile, the resource requirements of digital computing and deep learning are growing exponentially, in an unsustainable manner. One possible way to bridge this gap is the adoption of resource-efficient brain-inspired “neuromorphic” processing and sensing devices, which use event-driven, asynchronous, dynamic neurosynaptic elements with colocated memory for distributed processing and machine learning. However, since neuromorphic systems are fundamentally different from conventional von Neumann computers and clock-driven sensor systems, several challenges are posed to large-scale adoption and integration of neuromorphic devices into the existing distributed digital–computational infrastructure. Here, we describe the current landscape of neuromorphic computing, focusing on characteristics that pose integration challenges. Based on this analysis, we propose a microservice-based conceptual framework for neuromorphic systems integration, consisting of a neuromorphic-system proxy, which would provide virtualization and communication capabilities required in distributed systems of systems, in combination with a declarative programming approach offering engineering-process abstraction. We also present concepts that could serve as a basis for the realization of this framework, and identify directions for further research required to enable large-scale system integration of neuromorphic devices.

## 1. Introduction

The accelerating developments of digital computing technology and deep learning–based AI are leading toward technological, environmental, and economic impasses (Thompson et al., 2021; Mehonic and Kenyon, 2022). With the end of Dennard transistor-scaling (Davari et al., 1995) and the anticipated end of Moore's law (Waldrop, 2016; Leiserson et al., 2020; Shalf, 2020), conventional digital computers and clock-driven sensor systems face considerable hurdles regarding bandwidth and computational efficiency. For example, the gap between the computational requirements for training state-of-the-art deep learning models and the capacity of the underlying hardware has grown exponentially during the last decade (Mehonic and Kenyon, 2022). Meanwhile, in stark contrast, distributed digitized systems—ever-growing in size and complexity—require increasing computational efficiency for AI applications at the resource-constrained edge of the internet (Zhou et al., 2019; Ye et al., 2021), where sensors are generating increasingly unmanageable amounts of data.

One approach to addressing this lack of computational capacity and efficiency is offered by *neuromorphic engineering* (Mead, 1990, 2020). There, inspiration is drawn from the most efficient information processing systems known to humanity—brains—for the design of hardware systems for sensing (Tayarani-Najaran and Schmuker, 2021) and processing (Zhang W. et al., 2020; Basu et al., 2022) that have the potential to drive the next wave of computational technology and artificial intelligence (Frenkel et al., 2021; Christensen et al., 2022; Mead, 2022; Shrestha et al., 2022). Neuromorphic—that is, brain-like—computing systems imitate the brain at the level of organizational principles (Indiveri and Liu, 2015), and often also at the level of device physics by leveraging nonlinear phenomena in semiconductors (Chicca et al., 2014; Rubino et al., 2021) and other nanoscale devices (Zidan et al., 2018; Marković et al., 2020) for non-digital computation. The idea of using nonlinear physical phenomena for non-digital computing has been explored for decades. Different choices of underlying mathematical models lead to different definitions of what the concept of “computation” entails (Jaeger, 2021), and likely also influences the set of possible emergent innovations.

Here, we define neuromorphic computing (NC) systems as information processors in which the structure and function either emulate or simulate the neuronal *dynamics* of brains—especially of somas, but sometimes also synapses, dendrites, and axons—typically in the form of spiking neural networks (SNNs) (Maass, 1997; Nunes et al., 2022; Wang et al., 2022). NC systems feature asynchronous massive parallelism, sparse, event-driven activity, and co-location of memory and processing (Indiveri and Liu, 2015), open up new algorithmic spaces (Adam, 2022; Schuman C. D. et al., 2022), and offer superior solutions to a range of brain-like computational problems in terms of energy-usage and latency (Davies et al., 2021; Göltz et al., 2021; Stöckl and Maass, 2021; Yin et al., 2021; Rao et al., 2022). Furthermore, beyond cognitive applications, SNNs and NC systems have also demonstrated potential for applications such as graph algorithms, constrained optimization, random walks, partial-differential-equation solving, signal processing, and algorithm composition (Aimone et al., 2022). Consequently, there is a growing interest for NC technology within application domains such as automotive technology, digitized industrial production and monitoring, mobile devices, robotics, biosensing (such as brain–machine interfaces and wearables), prosthetics, telecommunications-network (5G/6G) optimization, and space technology.

One challenge facing neuromorphic technology is that of integrating emerging diverse hardware systems, such as neuromorphic processors and quantum computers, into a common computational environment (Vetter et al., 2018; John et al., 2020). Such hardware is likely to be increasingly included in computational ecosystems to facilitate or accelerate particular types of computation (Hamilton et al., 2020; Leiserson et al., 2020; Shalf, 2020), due to performance constraints of existing computational hardware in, for instance, energy usage or processing speed. Fundamental trends in computer-architecture development indicate that nearly all aspects of future high-performance computing architectures will have substantially higher numbers of diverse and unconventional components than past architectures (Becker et al., 2022), leading toward a period of “extreme heterogeneity”. Consequently, neuromorphic processors are, in many future use-cases, likely to be part of a broader, heterogeneous computational environment, rather than to be operated in isolation. Thus, there is a need for programming models and abstractions, as well as interparadigmatic communication principles and data models, that enable interoperability between NC systems and large-scale distributed systems of digital systems (Maier, 1998). In this article, we will use “digital computing (DC)" to refer to *conventional* computational technology based on the von Neumann architecture and synchronous logical processing, including conventional distributed computing and systems of systems.

Here, we address the technology gap of interoperability between NC and DC systems. Such interoperability requires an integration architecture and associated means for implementation, validation, and verification. An associated challenge is the different viewpoints and property understanding and terminology of the NC and DC communities, which we attempt to contribute to bridging with this article. We frame the addressed gap in terms of the following main challenges:

1. **Communication:** How to represent and transcode information between NC and DC systems to establish interoperability and enable efficient hybrid NC–DC systems?
2. **Virtualization:** How to provide seamless access to NC systems in distributed DC systems, with robust and trustworthy NC–DC interfaces?
3. **Programming:** How to program hybrid NC–DC systems efficiently?
4. **Testing and validation:** How to reliably train and test the functionality of hybrid NC–DC systems?

We outline the current landscape of NC technology from the perspective of system interoperability and integration, describing the most significant qualities of NC systems as compared to the fundamentally different DC paradigm. Based on this description, we outline a conceptual framework for integration of NC systems based on microservices. The framework consists of a neuromorphic-system proxy for providing virtualization and communication capabilities required in distributed settings, in combination with a declarative programming approach offering engineering-process abstraction. We present established concepts for programming, representation, and communication in distributed systems that could serve as a basis for the realization of this framework, and identify directions for further research required to enable NC systems integration.

In Section 2, we describe NC systems. In Section 3, we outline the conceptual integration framework and discuss relevant computer-scientific concepts. In Section 4, we discuss an example use-case of the framework. Finally, in Section 5, we summarize the presented work, and present some concluding remarks.

## 2. Neuromorphic systems

The field of neuromorphic engineering dates back to the late 1980s (Mead, 1990, 2020), and originally dealt with the creation and use of sensing and processing systems that imitate the brain at the level of structure and device physics. Today, the term “neuromorphic” has broadened, and “neuromorphic processors” typically refer to hardware systems of different architectures that are specialized for running spiking neural networks (SNNs). Neuromorphic hardware architectures thus range from electronic emulation with analog circuitry, or novel electronic devices, to digital systems specialized for massively parallel differential-equation solving for spiking neuron models. However, as SNNs (Maass, 1997; Nunes et al., 2022; Wang et al., 2022) are inherently event-driven, asynchronous, time-dependent, and highly parallel, all neuromorphic processors, by consequence, differ significantly from DC systems, as summarized in Table 1. In general, analog-based NC systems are more power-efficient than fully digital ones (Basu et al., 2022), by leveraging device physics for real-time neurosynaptic emulation, while digital systems come with the versatility of being fully configurable by logical programming. Due to the need for power-efficient sustainable technologies for AI workloads, neuromorphic solutions can come to constitute up to 20% of AI computing and sensing revenue by 2035^1^.

**Table 1:** Qualitative differences between conventional digital computing (DC) and neuromorphic computing (NC) architectures.

**Architecture**	**Conventional digital**	**Neuromorphic**
**Processing operations**	Sequential	Massively parallel
**Memory–processing organization**	Centralized, separated	Distributed, colocated
**Temporal organization**	Synchronous, clock-driven	Asynchronous, event-driven
**State qualities**	Discrete, static	Continuous, dynamic
**Programming method**	Sequential logic	Structural SNN configuration
**Unit of communication**	Binary numbers	Unary spike-events (spatiotemporal, sparse)

### 2.1. States in neuromorphic systems

The state of a neuromorphic processor at any given moment is defined by the properties of the SNN that processor has been configured to implement and the event-based neurosynaptic activity of that SNN, which is largely reactive in response to input signals. These properties can roughly be arranged into the following categories:

- **Structural properties:** For example network topology, synaptic weights, time constants, axonal delays, and neuronal thresholds.
- **Transient properties:** For example neuronal potentials, synaptic currents, and spiking activity.

Out of these properties, it is, in general, the structural ones that are subject to direct manipulation by external configuration, optimization, and learning algorithms. The transient state, on the other hand, rather arises in reaction to presented input signals in a way that is determined by the structural state. However, a clear line cannot simply be drawn between structural and transient properties, as the biological timescales of synaptic plasticity phenomena—that is, the changes in strength and structure of neuronal connections—range from single milliseconds, in the case of short-term plasticity, to the whole lifetime of an organism, in the case of structural plasticity (Jaeger et al., 2021). Many neuromorphic systems do, however, exclude on-chip implementation of synaptic plasticity due to the complexity and resource cost (Frenkel et al., 2021), in which case, structural parameters are more clearly distinguished as subject to configuration by an external system. There are many learning rules in use due to the knowledge gap associated with long-term plasticity and task-dependent requirements. Therefore, some neuromorphic systems implement flexible DC coprocessors for learning (Painkras et al., 2013; Davies et al., 2018; Grübl et al., 2020).

### 2.2. Information in neuromorphic systems

DC systems represent information in clock-driven discrete states, the resolutions of which are determined by the number of bits used for representing binarily encoded variables. NC systems, on the other hand, represent information using unary (one-or-nothing), uniform interneuronal spike-events. These carry explicit information about both space and time in their source of origin and time of arrival—potentially carrying arbitrary temporal precision in the interspike intervals (Thorpe et al., 2001). This form of representation arises already in neuromorphic sensors, as they rely on level-crossing Lebesgue sampling (Astrom and Bernhardsson, 2002) for event-driven generation of sense data, or, alternatively, in delta-modulated spike-data conversion of conventionally sampled signals (Corradi and Indiveri, 2015). Spike-timing-based representations thus allow asynchronous, sparse event-driven sensing and processing with lower sample complexity and capabilities beyond those of classical encoding and processing systems (Adam et al., 2020, 2022). Consequently, this enables energy-efficient systems, especially for real-time applications in which both sensing and processing are spike-based and event-driven (Liu et al., 2019).

#### 2.2.1. Neural code

There are several ways in which spatiotemporal combinations of uniform spikes could theoretically be used to encode information. Thorpe et al. (2001) outline the following theoretical spike-based neural coding schemes:

1. **Rate code:** Information is represented by *how often* each single neuron fires, in the form of a time-averaged firing rate. (ANNs are based on rate code.)
2. **Count code:** Information is represented by *how often* a group of neurons fire in total during a temporal interval.
3. **Binary code:** Information is represented by *which* neurons fire during a temporal interval. A binary sequence is formed from the array of neurons by viewing them as being in one of two states: active or inactive.
4. **Timing code:** Information is represented by *when and where* each spike occurs—that is, in the source of origin and time of arrival.
5. **Rank-order code:** Information is represented by the *temporal order* in which a group of neurons fire, but without further spike-timing information.
6. **Synchrony code:** Information is represented by *which* neurons fire closely in time to each other during a temporal interval.

This list is not exhaustive, and the manner in which information is actually represented in the brain is still largely an open question (Brette, 2015; Zenke et al., 2021). It is, for instance, possible that the asynchronous dynamics of SNNs give rise to emergent representations that consist of combinations of coding schemes such as those listed above. Nevertheless, the listed coding schemes—along with their estimated capacity for information transmission, see Table 2—provide an overview of the qualitatively distinct ways in which information can be represented in SNNs, and the quantitative relations between these.

**Table 2:** Information-transmission capacity of different neural coding schemes.

**Coding scheme**	**Possible states (no.)**	**Equivalent bits (no.)**	**Comments**
Timing code	(*t*/δ*t*)^*N*^	33	For temporal resolution δ*t* = 1 ms
Rank-order code	*N*!	21	Temporal ordering
Synchrony code	$n_{\Phi }^{N}$	20	For *n*_Φ_ = 3 possible phases
Binary code	2^*N*^	10	Used in conventional computers
Count code	*N*+1	3.46	Equivalent to rate code in this scenario

Adapted from Nilsson (2021). Arranged in descending order of capacity, as theoretically estimated by Thorpe et al. (2001). *N* is the number of neurons, each spiking maximally once. The number of equivalent bits were calculated for the example scenario of *N* = 10 and a temporal interval of *t* = 10 ms.

The estimates presented in Table 2 were made for a population of *N* = 10 neurons, during a temporal interval of *t* = 10 ms, and with a temporal resolution of 1 ms. As the estimations were made for scenarios of rapid processing, they were also limited to a maximum of one spike per neuron. During these conditions, rate code is theoretically equivalent to count code, as rate code would require more than a single spike per neuron—and thus a longer duration—to represent more information. While these estimates were made for limited conditions, it illustrates how the information transmission per spike would be maximal if spikes carried information in their precise timings. However, a spike-timing-based coding scheme may demand a high level of complexity and temporal precision in the decoding mechanisms. It is important to note here that the data generated by neuromorphic, event-driven sensors is, at least in part, intrinsically spike-timing coded due to the event-driven, sparse activations that underlie the low-power, low-latency operation of such sensors (Liu et al., 2019). Therefore, in order to gain analogous benefits in the subsequent processing, it is likely necessary to incorporate some degree of spike-timing code in neuromorphic processing systems. However, the choice of coding scheme is likely to be task-specific and subject to optimization (Guo W. et al., 2021; Forno et al., 2022; Schuman C. et al., 2022).

#### 2.2.2. Representation space

**Figure 1:**
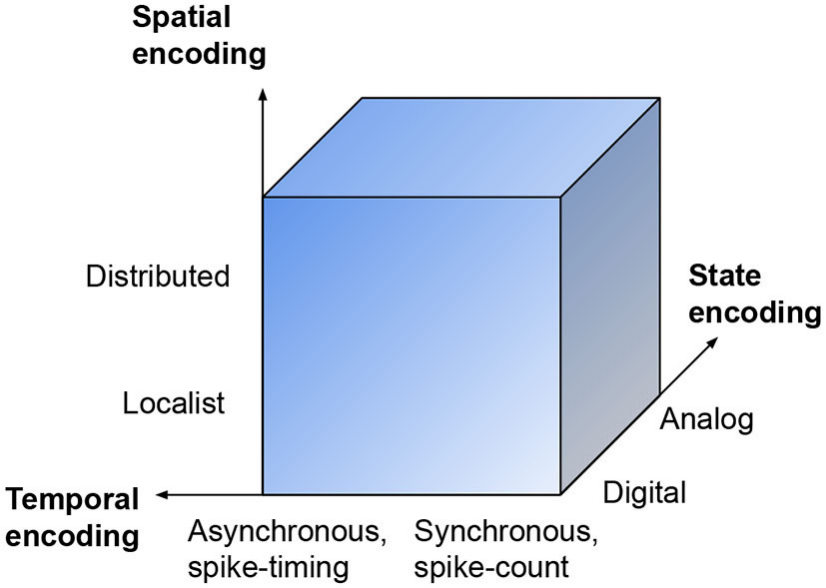
Space of possible representations in neuromorphic systems. The illustration depicts a continuum, in which gradual, independent changes along each dimension are possible, as there exists many variations and combinations of the mentioned concepts.

Figure 1 illustrates a conceptualization of the space of possible information representations in NC systems. As discussed previously, the NC hardware substrate may, to varying degrees, rely on digital or analog circuitry, and the temporal encoding may, again to varying degrees, asynchronously rely on the precise timings of spikes in qualitatively different coding schemes, see Table 2. The spatial dimension of a neural network is generally, by default, used for *distributed representations*, in which the representations of different concepts are distributed over several of the same neurons and synapses of the network. Conversely, *localist representations*, which are studied in conventional, logical neurosymbolic computation (Garcez and Lamb, 2020; Dold et al., 2022), represent different concepts with single, discrete identifiers, such as single neurons or bits. An example of a completely localist representation could, for instance, be a single “cat neuron,” which, when activated, signifies the inferred presence of a cat in sensory data. As in the case of the two other dimensions of Figure 1, it also depicts a possible spectrum of spatial encoding, in the hypothetical extremes of which, representations are either distributed across a whole neural network or localized to single neurons, respectively.

#### 2.2.3. Interfaces and semantics

For the activities of a neural network to ultimately have discernible implications for action in an external system, *symbolic representations* are needed, such as for example: “Pattern A implies Action X” and “Pattern B implies Action Y.” “Action” is here meant in a broad sense, ranging from read-out signaling to motor-output actuation. In the kind of hybrid NC–DC systems discussed here, such symbolic representations would, depending on the application, not necessarily have to be generated within the confines of the NC system itself. However, at every system interface, there needs to be an operational *semantic*—a system of interpretation—for transmitted data (Nilsson and Sandin, 2018; Nilsson, 2022). Such interfaces could, for instance, be implemented in the form of microservices, which are further discussed in Section 3.

An interesting concept for interface semantics for NC systems is that of *embeddings*—a widely used form of representation in neural networks and machine learning that is essential to many state-of-the-art models. Embeddings are succinct, intermediate representations of associations within usually large-scale datasets. For example, embeddings are used in deep learning architectures in which concept representations are quickly learned from combinations of sensor data, descriptive sentences, and higher-level knowledge representations (Mei et al., 2022). The learned concepts can be used in downstream applications, such as answering questions by reasoning about unseen sensor inputs. Typically, embeddings emerge as nested function values in a deep learning model, *y*_*i*_ = *f*_*n*_(…*f*_0_(*x*_*i*_; θ_0_);θ_*n*_). The parameters θ_*k*_ are optimized to provide the outputs *y*_*i*_ that are expected for the corresponding inputs *x*_*i*_ with minimum error according to some metric. The embeddings, *f*_*k*_(…), of a model that is optimized on a sufficiently large and varied dataset are often useful for other similar datasets, as these embeddings can be used as input to optimize a new output function with less data and computational resources compared to full model retraining. Embedding are used in various models—autoencoders, transcoders, multimodal predictive models, etc.—and are natural components to be used and supported by the envisioned hybrid neuromorphic–digital computing systems and programming models. This entails interesting opportunities and challenges related to the optimization and interoperability of embeddings realized in different hardware and all the related symbols appearing in software and data across an orchestrated system (Nilsson et al., 2019, 2020).

### 2.3. Programming of neuromorphic systems

In contrast to DC systems, the actions of NC systems are not primarily dictated by sequences of explicit logical instructions. Rather, the analog to DC programs in NC systems can be considered as being implicitly defined by the structural properties, as defined in Section 2.1, of the implemented SNNs, as these determine the input–output signal transformations that are performed. Furthermore, while DC systems encode information in observable, static, discrete states, NC systems may inhabit unobservable, dynamic, continuous states, and they receive inputs in the form of uniform spike-events, in which information is encoded in the physical time of arrival and source of origin. Thus, the use of NC systems is fundamentally different from that of DC systems, and—in order to fulfill the potential for efficiency—may require a significant change of perspective in the view of programming (Schuman C. D. et al., 2022) and computation (Jaeger et al., 2021) informed by neuroscience and dynamical systems theory.

Some attempts to develop programming abstractions for NC systems include the Neural Engineering Framework (NEF) (Stewart, 2012) and Dynamic Neural Fields (DNFs) (Sandamirskaya, 2014). However, these are often fairly limited to specific use-cases—biologically plausible neural models for the NEF, and models of embodied cognition for DNFs. Thus, there is a gap in defining more generally useful programming abstractions for neuromorphic computing systems (Schuman C. D. et al., 2022), including virtualization concepts required for seamless edge-to-cloud integration.

#### 2.3.1. Software

As the landscape of neuromorphic computing is made up of different hardware and software architectures that are developed by different groups, it is characterized by a fragmented and noncomposable array of programming models and frameworks. Programming frameworks for SNNs and neuromorphic hardware generally fall into one of the following categories:

- **Optimization tools:** SNN-parameter optimization tools, usually based on supervised deep learning, such as SNN Conversion Toolbox (Rueckauer et al., 2017), SLAYER (Shrestha and Orchard, 2018), Whetstone (Severa et al., 2019), EONS (Schuman et al., 2020), and EXODUS (Bauer et al., 2022).
- **Simulators:** SNN simulators with low-level APIs for conventional computers, such as NEST (Gewaltig and Diesmann, 2007), Brian 2 (Stimberg et al., 2019), Nengo (Bekolay et al., 2014), GeNN (Yavuz et al., 2016), BindsNET (Hazan et al., 2018), Rockpool (Muir et al., 2019), SINABS ^2^, and Norse (Pehleand Pedersen, 2021).
- **Hardware interfaces:** Low-level interfaces and runtime frameworks for configuration of neuromorphic hardware, such as PyNN (Davison et al., 2009), Fugu (Aimone et al., 2019), Samna ^3^, BrainScaleS OS (Müller et al., 2022), and Lava.

While several frameworks exist, none of them have so far provided programming abstractions that are composable and span the diverse range of algorithms and methods within NC (Davies et al., 2021; Jaeger, 2021). Furthermore, NC hardware typically have limitations in terms of connectivity, plasticity, and neurosynaptic configurations. Thus, transforming a well-defined SNN specification or general program into a corresponding hardware configuration is challenging and further complicated by imperfections of mixed-signal circuits and limited resources, such as bandwidth, which generate differences from the specified target. Even between generations of the same hardware architecture, such as Spikey and BrainScaleS 1, SpiNNaker 1 and 2, or Loihi 1 and 2, it is often difficult to build upon existing software (Müller et al., 2022). As of today, two candidate models for general-purpose and platform-agnostic NC configuration are PyNN and Lava.

##### 2.3.1.1. PyNN

PyNN (Davison et al., 2009) is a simulator-agnostic language for describing SNN models at the level of network topology, neurosynaptic parameters, plasticity rules, input stimuli, and recording of states, while still allowing access to the details of individual neurons and synapses. PyNN also provides a set of commonly used connectivity algorithms (e.g., all-to-all, random, distance-dependent, small-world) but makes it easy to provide custom connectivity in a simulator-independent way. PyNN provides a library of standard models of neurons, synapses, and synaptic plasticity, which have been verified to work the same way on the different supported simulators. As of today, common SNN simulators and some hardware emulators support PyNN, which is also the entry-point to the BrainScaleS and SpiNNaker systems that implement PyNN as an experiment-description language.

##### 2.3.1.2. Lava

Lava^4^ by Intel is an upcoming open-source software framework for neuro-inspired applications and their deployment on neuromorphic hardware, and constitutes an attempt to move toward convergence in the domain of NC software. Lava is designed to be hardware-agnostic, modular, composable, and extensible—allowing developers to construct abstraction layers to meet their needs, and to broaden the accessibility of programming NC systems. The fundamental building-block in Lava, for algorithms and applications alike, are so-called *processes*—stateful objects with internal variables and input and output ports for message-based communication *via channels*. This architecture is inspired by the communicating sequential processes (CSP) formal language for asynchronous, parallel systems, which belongs to the family of formal models for concurrent systems known as *process calculus*, further described in Section 3.2. Every entity in Lava—including neurons, neural networks, conventional computer programs, interfaces to sensors and actuators, and bridges to other software frameworks—is a process with its own memory and message-based communication with its environment. Thus, Lava processes are recursive programming abstractions, from which, modular, large-scale parallel applications can be built.

### 2.4. Challenges to adoption and integration

The following are some of the major challenges posed to the adoption of NC technology and its integration into the present computational environment.

#### 2.4.1. Programming abstractions and frameworks

As discussed in Section 2.3, there is a lack of common programming abstractions, models, and frameworks for different NC designs (Davies et al., 2021; Schuman C. D. et al., 2022). Intel's launch of the Lava software framework is an attempt at closing this gap, but, being so recent, the degree to which Lava will aid in achieving NC software convergence remains to be proven. Furthermore, there may be a need for further developments of generalized system hierarchies, concepts of completeness (Zhang Y. et al., 2020), and analytical frameworks (Guo Y. et al., 2021) for NC systems and other unconventional computing concepts (Jaeger, 2021), to facilitate hardware–software compatibility, programming flexibility, and development productivity.

#### 2.4.2. Interdevice communication

Most neuromorphic systems—sensors and processors alike—implement an address-event representation (AER) spike-event communication protocol (Mortara and Vittoz, 1994; Boahen, 2000), in which events, signified by the address of their source of origin, such as a neuron or pixel, are asynchronously generated and transmitted in real-time along the connections of neural networks. However, although it is standard practice to implement *some* AER protocol, there are slight differences in the implementations between the different neuromorphic sensory and processing devices that currently exist (Basu et al., 2022), as these are developed by different groups. This discrepancy between different neuromorphic devices impairs their interoperability, as well as standardization of NC–DC communication, and thus poses a challenge to the integration of neuromorphic systems into the broader computational environment.

#### 2.4.3. Reliance on host computers

Currently, the use of NC systems relies heavily on conventional host computers for software deployment and, often, for communication with the environment *via* sensors and actuators. This reliance on a host machine—which performs preparation and deployment of the NC model and pre- and post-processing of spike-data—can impact the resource requirements for running the neuromorphic system to such an extent that the performance benefits of using such specialized hardware are lost (Diamond et al., 2016). Thus, there is a need to optimize the host–device communication architecture with regard to scalability, throughput, and latency, as well as to design and implement SNNs and NC systems in a way that minimizes the need for host–device communication in the first place.

## 3. Conceptual integration framework

The demand for computing keeps increasing due to the high value of digitization, and recent enabling technology trends such as cloud computing, IoT, extended reality, and AI (Gailhofer et al., 2021; Thompson et al., 2021). The future of industry and society will be shaped by opportunities to extract values from the corresponding huge volumes of data generated at the edge of the network, and to provide resource dynamism and scalability across the cloud-to-edge computing continuum. In general, this development involves challenging computational problems such as sequence learning, federated learning, and constrained optimization. Conventional DC technology alone is not sufficient to address such computationally intensive problems under tight energy and latency constraints in resource-constrained application domains such as battery-driven sensors and mobile devices. The computing performance can be improved by orders of magnitude, in factors of energy and latency, by combining NC and DC technologies in domain-specific configurations, see Davies et al. (2021) and Kugele et al. (2021) for examples. Thus, NC and DC technologies need to coexist in an increasingly heterogeneous cloud-to-edge computational environment (Hamilton et al., 2020; Shalf, 2020), to provide the required strong data processing capacity at the edge of the network as well as robust dynamic resource provisioning in the cloud-to-edge continuum. These challenges and technological trends require a corresponding advancement of next-generation software capable of enabling interoperability between fundamentally different computational architectures, such as NC and DC.

The problem of efficiently and effectively integrating NC into the existing and more traditional digital environment is not a trivial task for several reasons. The aforementioned challenges associated with adopting NC technology (see Section 2.4) are combined with new challenges related to integrating the two different computational paradigms. Some of the new integration challenges lie in the differences between the information representation and data models, the difficulties involved in programming non-deterministic and partially opaque NC systems with continuous dynamics, and the communication between the two types of technologies. The interpretation, transformation, and communication of information between DC and NC systems are key steps toward interoperability and full integration. Therefore, a DC-compatible conceptual framework that allows the community to investigate and address these challenges is needed. Such a framework needs to be sufficiently specific to allow co-existence and integration with existing DC systems, infrastructure, and software engineering processes, while also being sufficiently general to allow the performance benefits of unconventional information processing principles in NC systems.

In this section, we outline concepts that we consider important for the design of such a framework, and we discuss well-established methods and tools that may be helpful to address some of the associated challenges. In particular, we discuss the roles of data models, communication, and declarative programming from a conceptual viewpoint, thereby contributing a description of the knowledge gap between the NC literature and the paradigm of *microservices* (Larrucea et al., 2018), which underlies modern distributed DC systems. Microservices are small, single-responsibility applications, inspired by service-oriented computing, that can be deployed, scaled, and tested independently, and which can be combined at run-time to perform complex tasks and provide robustness in terms of, e.g., failover and strong security. Considering the aforementioned efficiency improvement needs, broad adoption of NC technologies is motivated, and this will require a combination of best practices for designing robust and efficient distributed systems—using microservices—and the unconventional information processing characteristics of NC systems.

**Figure 2:**
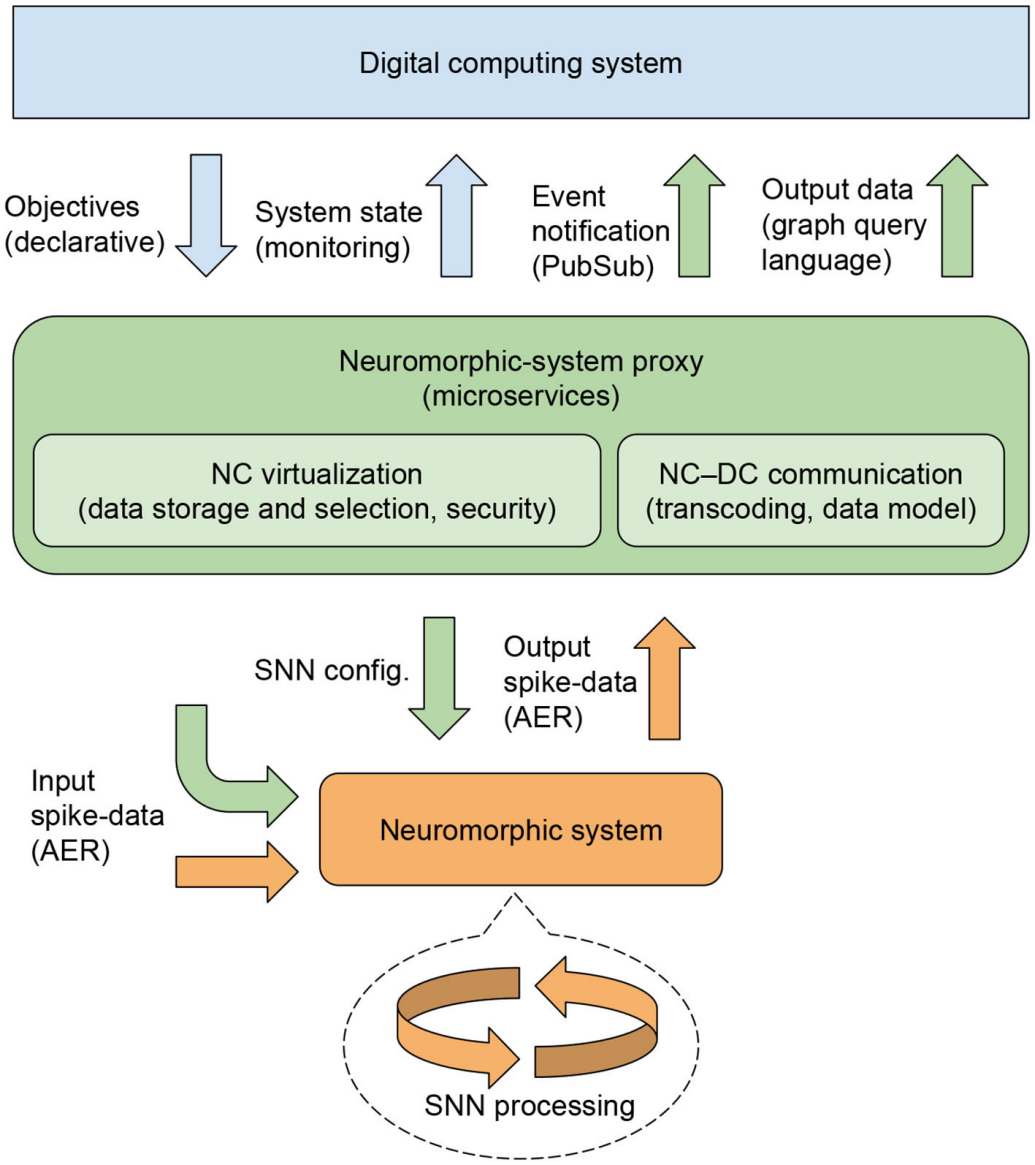
Conceptual system integration framework for neuromorphic devices in digital–computational infrastructure. Arrows symbolize information channels. The coloring indicates information being represented and transmitted within the following domains: orange: spike-based neuromorphic sensing and processing (NC); green: a virtual neuromorphic-system proxy; blue: conventional digital computing (DC).

The proposed framework, see Figure 2, consists of NC–DC abstraction layers and communication channels that we consider to be necessary for such an NC-system integration. Specifically, the digital abstraction is provided by a microservice instance, called a neuromorphic-system proxy (NSP), illustrated in the middle box of Figure 2, which constitutes a virtual representation and interface to a physical NC system, see the bottom box in Figure 2. The role of the NSP would be to provide virtualization and data mapping between the NC and DC domains, thereby providing efficient interfacing between them and managing, for instance, availability and security. The use of a microservice architecture provides characteristics such as late binding, loose coupling, and discovery to the framework. In addition to these characteristics, a major benefit of the use of microservices in the NSP is service longevity, which means that services are available over time. In contrast to the NC systems that provide transient data in real-time reaction to events, the NSP stores relevant data communicated from the NC system for access *via* the exposed microservices.

Building on service-oriented architecture (SOA)/microservice best practices (Erl, 2007; Delsing, 2017; Baškarada et al., 2018), the NSP would be software-defined and typically built on a library of base-software images (e.g., docker images) stored in a repository to provide easy instantiation and replication at the edge or cloud as desired. Application-specific programs would be added as delta images. Although Figure 2 shows a one-to-one mapping between an NC system and an NSP, this model could in principle be scaled to arbitrary numbers of NC systems and NSPs. The NSP is here described by first covering the essential interfaces, i.e., the services provided or consumed and then, in Section 3.1, some challenges and approaches for data modeling and mapping are described. The interfaces to the NSP are represented by arrows in Figure 2. The bottom part of the figure illustrates the following interfaces between the NC system and the NSP:

- **SNN configuration:** Drives the SNN configuration as needed for specific use cases. This is relatively slowly changing data. Initially, we envision the configuration to be primarily static, while it may be somewhat dynamic to allow some degree of digital reconfiguration as part of normal runtime operation.
- **Input spike-data:** The input to the SNN, in which most data (spikes) typically come from other neuromorphic systems such as sensors. In addition, input data from other systems can also enter through the digital interfaces of the NSP. All incoming spikes are processed by the NC system with the objective to produce an output that aligns with the requirements expressed by the declarative definitions and the validation protocol.
- **Output spike-data:** Involves information from selected spike-sources, such as specific read-out neurons (as defined by the SNN config.). Typically, abstractions are formed such that only spikes from neurons representing high-level information, such as in the last layers of a deep SNN, are communicated to the NSP. This serves to reduce the load of events coming to the NSP, although the transmitted data is still highly dynamic.

The top part of Figure 2 illustrates the following interfaces between the DC system and the NSP:

- **Objectives:** Interface for adding declarative statements that will be translated to an SNN config. This is complementary to code that is part of the NSP image. The need depends on the dynamics of reconfiguration required, and using it is therefore optional.
- **System state:** Interface for retrieving the SNN configuration in terms of structural SNN properties, see Section 2.1.
- **Event notification:** Interface based on publish–subscribe (PubSub) messaging (see Section 3.1.2), in which events are sent from the NSP. In alignment with the microservice paradigm, these events are typically sent to some event-handlers using different channels or topic trees to notify subscribers effectively and efficiently.
- **Output data:** Interface where other systems can request additional data related to the events seen in the event notification or related to any history or state stored in the NSP. This is basically a restful interface, but aiming for a graph query language, in which there is a well-defined typed data schema and where under- or over-fetching is avoided.

Within the NSP, a virtualized representation of the NC system (a digital twin) would provide key functionality to allow for representations of the NC system to exist on one or more DC systems. The virtualization component also provides the essential functionality of data selection and storage that makes it possible to aggregate and select which data from the NC system should be exposed to the digital part of the system. This component also provides storage, allowing access to data *a posteriori* to its creation— either through short-term caching, which allows efficient access for multiple clients, or longer-term storage for historical access to data.

In the subsequent parts of this section, we discuss established computer-scientific concepts with the potential to realize different parts of this framework or, if insufficient, that could inform the extension or development of new such concepts.

### 3.1. Communication and data models

In order to integrate NC systems with other entities in distributed DC systems, the standardized protocols and data structures used for information representation and communication in the DC domain need to be interfaced within the NSPs. This is a challenging problem considering the different information representations used in the DC and NC domains, including for example state-based and time-based representations, differences in semantics, and a variety of information encoding and transcoding methods used. Here, we review some well-established DC concepts that share characteristics with the NC domain, and may thus be helpful for addressing the challenges of data modeling and information translation and representation.

#### 3.1.1. Soft-state models

The event-based nature of NC systems maps well to the best-effort message-passing based nature of many current computer networks, such as the Internet. A key feature for best-effort computer networks to provide a distributed system state across a network, in a scalable manner that is robust to network disruptions and do not require too much communication overhead, is the concept of *soft state* (Ji et al., 2007). This is used in Internet routing protocols and other networked distributed systems and becomes even more important in more unstable systems such as *ad hoc* or delay tolerant networks (Perkins et al., 2003; Fall and Farrell, 2008; Grasic et al., 2011).

In a soft-state system, a distributed state is maintained in individual nodes by periodic update messages (or *events*) that are sent by other nodes in the network to refresh the current state. Such state can for example be used to maintain routes through the network for forwarding data traffic. If no refresh messages have been received within a certain amount of time, it is assumed that the state of the system has changed (e.g., a neighboring node has moved away and can no longer be reached), and thus the local state is removed. Since the underlying network is best-effort and refresh messages can be lost due to contention or interference, the system might wait until more than one expected refresh message has not been received before determining that the local state should be removed. The threshold used to determine a state change depends on the expected characteristics of the network and is a tradeoff between how rapidly the system reacts to state changes and the desired robustness to temporary disruptions. The benefit of utilizing soft state is that there is no risk of stale state information becoming stored indefinitely in parts of the network that cannot be reached by state-removal messages, and the need for such control traffic is removed completely.

The soft-state model is similar to NC systems in that messages (spikes) trigger and maintain states in nodes (neurons). Thus, we believe that there are potential benefits from mapping these models in the NC domain to digital integration in terms of data models.

#### 3.1.2. Event notification

The Internet has enabled the creation of very large distributed systems, commonly with the purpose of sharing information between geographically distributed data producers and consumers. In such systems, the publish–subscribe (PubSub) messaging paradigm, based on event notification and messaging, has received much attention for the loosely coupled form of interaction it provides in large-scale settings (Liu and Plale, 2003). In this paradigm, subscribers register their interests in a topic or a pattern of events and then asynchronously receive events matching their interest. PubSub provides decoupling in space (interacting subscribing and publishing parties do not need to know each other), time (parties do not need to interact at the same time), and asynchronous communication (subscribers can get notifications immediately when published) (Eugster et al., 2003).

PubSub systems can be categorized into subject-based, aka topic-based, and content-based (Liu and Plale, 2003). Subject-based PubSub means that a subscription targets a group, channel, or topic, and the user receives all events that are associated with that group. With content-based PubSub systems, subscriptions are instead based on queries or predicates, based on which the decision of to whom a message is directed is made on a message-by-message basis. The advantage of a content-based system is that the subscriber can be provided with the needed information only and does not need to learn a set of topic names and their content before subscribing.

The performance of a content-based PubSub network is typically challenged by the expensive matching cost of content messages. Hybrid schemes between these types of PubSub systems exist, where subscribers register content-based subscriptions to one or more topics. For example, HYPER minimizes both the number of matches inside the PubSub network and the delay to receive subscribed content (Zhang and Hu, 2005). The hybrid schemes, aka type-based, thus represent the middle-ground between coarse-grained, topic-based systems and fine-grained, content-based systems. Type-based systems gives a coarse-grained structure on events (like in topic-based) on which fine-grained constraints can be expressed over attributes (like in content-based) (Shen, 2010).

PubSub systems based on event notification and messaging have been adopted by the SOA model (Perrey and Lycett, 2003; Levina and Stantchev, 2009) and its later incarnation known as the Application Program Interface (API) and microservice paradigm (Di Francesco, 2017). In contrast to traditional SOA, the API and microservice paradigm builds on the requirement that such services must be independently deployable (Xiao et al., 2016). Modern messaging systems are typically based on message-oriented middleware like Apache Kafka, Rabbit MQ, NATS Streaming, Google Pub/Sub, Microsoft Event Hubs and Amazon Kinesis (Eugster et al., 2003).

The properties provided by PubSub and event notification as used in SOA and microservice-based systems, i.e., space, time and synchronization decoupling, together with the ability to efficiently monitor states and outcome of NC systems, can prove useful to make NC systems interact efficiently with DC systems. For example, event notification can be used to signal a state occurrence in an NC system that motivates further investigation based on analysis of complementary output data, possibly involving requests to multiple services and systems. The problem of efficiently performing such requests requires a flexible query mechanism, for instance, an adaptation of a modern graph query language, which is discussed below.

#### 3.1.3. Information querying

In the DC paradigm, graph query languages allow users to make queries in databases and information systems based on *graph structures* (Wilson, 2010). Graphs can allow for well-structured data, with complete and understandable descriptions, from which precise fetching can be made, returning an appropriate sparse subset of the data. In the envisioned NC–DC hybrid systems, a similar query-based approach is needed to enable flexible and efficient access to functionality and data from the NC to the DC domain. One prominent example is the graph query language GraphQL^5^, which allows applications to get the required data in a single request that makes efficient use of network resources. GraphQL also allows the definition of new data fields and types without invalidating existing queries, which results in effective code maintenance. Similarly, graphs can be useful to describe and query neuromorphic system configuration and SNN state information, as well as SNN models, data models, and mappings between information representations in the NC and DC domains.

#### 3.1.4. Synthesizing a data model

One of the key success factors in NC–DC integration is to find a data model for the NSP that is suitable for both the NC and DC domains. The goal is to have a data model in the NSP where data can be filled out based on NC processing according to declarative instructions, and then effectively be provided to DC systems through the digital interfaces.

The precise definition of such a data model would be highly application-dependent. The goal within the outlined integration framework in this context is the existence of a generic foundation providing graph-oriented data modeling, with a well-defined syntax for defining the exact data model for each application case. This application-specific model would be set through the configuration interface of the NSP, and/or by software that defines the application-specific NSP. The same configurational information would also be used in concert for *SNN config*. This is to facilitate that the output spike-data from the NC system can be used by the NSP to fill out data in accordance with the data model.

In response to specific NC output spike-data, such as upon recognition of a monitored pattern, the NSP could trigger a digital event notification (see Section 3.1.2) to interfacing DC systems. Such a digital event could be triggered directly by certain NC spike-activity, such as from specific read-out neurons, or upon spike-data decoding and data update in the NSP according to internal criteria—see Section 2.2.3 for a discussion of NC interfacing and semantics. Subscribers of such events could, upon receiving interesting event notifications, reactively access more information by placing a graph query to the NSP to fetch desired parts of the graph data.

Many of the challenges in synthesizing a data model come from the fact that state models of the NC and DC domains are fundamentally different, while they are also complementary to some extent:

1. NC systems have continuously evolving transient states in response to input data (see Section 2.1), which give rise to more or less opaque patterns of spiking activity. The output spike data from the final neuron layer of an NC system should be decoded or interpreted according to some semantic and used to update the data model of the NSP. How this is to be accomplished is a subject for future research.
2. In the DC domain, in contrast to NC, states are typically not continuously decaying. Therefore, it would be possible to store abstract representations of the NC system state over time in the NSP. This is a complementary property to the NC system. However, the proposed kind of NSPs would consist of microservices typically operating close to the physical NC systems in an edge cloud, in which storage space is limited. To mitigate this limitation, such storage could be limited to snapshotting in response to interesting events, such as NC pattern recognition, as identified in the NSP spike-data decoding or read-out according to internal definitions. Furthermore, the maintenance of the NSP state could be made dependent on some form of triggering, such as repeated NC pattern recognition, and a soft-state protocol (see Section 3.1.1) could be used to remove outdated NSP states, which could instead be offloaded asynchronously to central clouds.

### 3.2. Declarative programming

This section discusses declarative programming as a goal-oriented approach to configuring NC systems that could enable engineers with commonly available competence to implement machine learning and AI solutions (Molino and Ré, 2021). Declarative programming is a programming paradigm in which the logic of computations is described without describing their control flow. In a declarative language, what is described is the goal a given program is intended to achieve (i.e., the desired outcome) rather than an explicit description of the sequence of computational primitives the program is to carry out. The promise and rationale of declarative approaches is thus that the focus is on describing “what,” not “how.” Advantages include clarity and unambiguity of the objectives, potential for automation by AI of the dynamic properties and placements within those objectives, and that correctness can be checked against the declarative objectives using a validation procedure. The declarations are free from imperative details—the “how”—and associated side effects. This is a suitable approach for NC systems, since there is no predefined control flow, and no desire to engineer traditional control flows.

Machine learning models, AI objectives, and NC system configurations are naturally defined in declarative languages (Schmitt et al., 2017; Gould et al., 2022), while the details of “how” are subject to optimization, search, and plasticity. Over the last years in deep learning, there has generally been a shift in modeling preferences, from a focus on neural-network-centric modeling, such as with TensorFlow (Abadi et al., 2015) and PyTorch (Paszke et al., 2019), toward higher-level interfaces supporting modern machine learning methods and tasks, such as TensorBoard for TensorFlow, Ludwig (Molino et al., 2019), and Weights and Biases (Biewald, 2020). Declarative deep learning frameworks (Molino and Ré, 2021), such as Ludwig, are aimed at facilitating a new generation of systems that are more user-friendly by focusing on defining the data-schema and tasks, rather than low-level neural network information. However, while ANNs and deep learning techniques are partly brain-inspired and offer a valuable starting point for development of NC algorithms and SNN models, they represent only a subsection of the space of spike-based neural computation (Roy et al., 2019) that is available to neuromorphic hardware (Indiveri and Sandamirskaya, 2019) and hybrid SNN–ANN models (Zhao et al., 2022), see Figure 1 and Table 2. Rather, NC abstractions need to be co-designed (Schuman C. D. et al., 2022) with the digitization requirements and NC modules to provide a seamless edge-to-cloud integration and overall efficient hybrid AI solutions (Zhao et al., 2022) to the general computational problems defined in use cases. Furthermore, constraints associated with the interfaces between the declarative programming level and SNN/ANN descriptions based on existing software frameworks (Qu et al., 2022) need to be considered.

In a declarative NC system, the desired outcomes would be used to partly autogenerate the system configuration using a set of established NC algorithms and SNN architectures, as well as to verify the compliance of system results. The outcomes would depend on the correctness of the declarative statements, the model and algorithm capabilities, and the data fed to the system. This leads to a need for verification and validation that resembles traditional software testing. However, the compliance of such declarative NC systems would not be totally deterministic as is typically the case in traditional declarative programs, in which the outcome can be verified logically and deterministically. Thus, the outcome of a declarative NC system for a given dataset would rather need to be validated statistically. We propose, for conciseness, to denote this entire process of statistical outcome-evaluation with respect to declarative statements and input data as a *validation procedure*, rather than a testing procedure, to emphasize the difference to traditional software testing. By that, we also avoid confusing the validation procedure with the testing of neuromorphic circuits, e.g., to detect manufacturing defects and faulty circuits, and runtime failures (Gebregiorgis and Tahoori, 2019; Hsieh et al., 2021).

#### 3.2.1. Test and validation methodology

As mentioned above, the verification, validation, and testing based on declarative statements and data fed to the NC system is here referred to as a validation procedure. This procedure aims to assess the statistical reliability of the NC system in meeting the declarative statements in the context of its structural and transient properties. As discussed in Section 2.1, the structural properties are subject to direct manipulation by external configuration, optimization, and learning algorithms, which would ideally be defined using declarative statements. The transient properties, on the other hand, depend on the data momentarily presented to the system and its current structural properties. Note that the validation procedure would mainly address a higher layer of abstraction than that of the transient SNN properties. In addition, given the dependence on input data, the transient state of a system that can be observed through a validation procedure may exist only in direct connection to when the data is presented to the system, while structural states may be observable at any time.

The validation procedure should focus on the system's capacity to *perform* the task learned, and, as such, it can also be useful in training protocols. Given that the inherent states of an NC system are updated at different time-scales, we believe that a validation procedure should aim to examine the system focusing on relevant use-case-specific time-scales. In this context, structural states should be examined to determine whether the NC system demonstrates the desired capacity to *converge* to a solution or *learn* a given type of task according to declarative statements, provided that the input data contains the needed information.

#### 3.2.2. Process calculus

Process calculus is a category of computer scientific approaches for the formal modeling of concurrent systems, which may serve as a base for declarative definitions of desired outcomes for networked NC systems. A process calculus provides a framework for high-level description of interaction, communication, and synchronization between independent processes or agents. Communication between processes is accomplished through channels, which can forward events that include some data. Many processes can send and/or receive from channels, so that complex distributed connectivity can be modeled, and there can be many processes on both the sending and receiving end of each channel. Channels are first class objects, i.e., they can be passed around as data on other channels to achieve dynamic connectivity between processes.

In the context of NC systems, there are new challenges to using process calculus related to the non-deterministic stochastic behavior of such systems, and to the fact that system validation depends on both the declarations and the input data. Nevertheless, there exists work tying process calculus to spiking neural systems (Ciobanu and Todoran, 2022), and Intel's NC software-framework Lava is inspired by the process calculus communicating sequential processes (CSP), see Section 2.3.1.

## 4. Discussion

### 4.1. An example use-case

In order to make the outlined conceptual integration framework more concrete, it may be useful to discuss it in terms of an example use-case. One relevant application area is vibration-based condition monitoring of rotating machinery for predictive maintenance by error or anomaly detection (del Campo and Sandin, 2017; Martin-del Campo et al., 2021), which has been investigated with SNNs in recent work (Dennler et al., 2021; Zuo et al., 2021). In such an edge-computing scenario, an NC sensory–processing system would be deployed in the rotating machinery, or close to functionally critical components such as bearings, where the lack of wired connection to the outside world makes the need for resource efficiency high to allow self-sustainable operation or a long battery life exceeding the component service interval. The NC system would be performing fault or anomaly detection by processing vibration sensor data in a reactive, event-driven manner. The sensor data would ideally be generated with a fully event-driven vibration sensor, with application-specific filter banks providing a succinct spike representation of relevant information.

Ideally, the NC system would be configured with declarative objectives *via* the *objectives* interface, with a minimal amount of explicit SNN configuration. Following the declarative objectives, an autogenerative SNN configuration would ensue by querying a set of established SNN models and algorithms for training, optimization, and/or pretrained model transfer, which would be applied *via* the *SNN config*. interface. Such a low-effort NC configuration would be dependent on the future development of NC programming models and abstractions, as well as an appropriate query language. Information about the current configuration of the NC system would also be communicated back for monitoring in an orchestrating DC system *via* the *system state* interface. Upon fault detection—signified, for instance, by threshold-crossing activity in specific read-out neurons or by some other form of spike-data decoding in the NSP—the NSP would notify subscribers in the distributed DC system about the occurrence of the fault *via* the *event notification* interface. Ideally, the subscribers would then be able to request more information retained in the NSP about the characteristics of recent sensory–processing data *via* the *output data* interface. This feature would be heavily dependent on the development of a *data model* and *transcoding* methods for such NC–DC communication, which are subject to future research. Furthermore, the *objectives* interface could be used to update the declarative objectives in order to reconfigure or refine the configuration of the deployed NC system.

### 4.2. Conclusion

In this work, we have addressed the technology gap of interoperability between NC and DC systems. We have described the current landscape of NC technology, focusing on aspects expected to become increasingly relevant for large-scale adoption and integration of NC systems into the present distributed DC infrastructure. Based on this analysis, we have proposed a conceptual microservice-based framework for NC systems integration, consisting of a neuromorphic-system proxy (NSP) that would provide virtualization and communication capabilities, in combination with a declarative programming approach that would offer engineering-process abstraction. We have also presented well-known concepts in computer science that could be combined as a basis for the realization of the proposed framework. We have identified the following main knowledge gaps, which may form a basis for future research:

- **Neuromorphic-system proxy:** A central question for the NSP is how to virtually represent the capabilities and states of physical NC systems, such that reliable microservices can be provided. Such representations need to balance level of detail with computational expense. For instance, a full virtual simulation of the neuronal dynamics and event communication within an NC system may be motivated at times, while not feasible to be constantly maintained.
- **Communication and data models:** In the proposed framework, NC–DC communication requires the development of an interparadigmatic data model that allows fetching relevant information from physical NC systems beyond the sparse event notifications and latent dynamic states. This is closely tied to the challenge of developing transcoding principles and operational semantics for interfacing with the spike-based representations of NC systems. Case studies focusing on optimization of spike encoders indicate that the best performing solutions are application-specific, which suggests that generic solutions require a combination of semantic technologies and machine-learning-based optimization.
- **Declarative programming:** A generic NC-programming framework requires appropriate programming abstractions, which enable engineers with commonly available competence to implement machine learning and AI solutions. Declarative programming, focusing on “what” rather than “how,” is a natural approach to describe learning objectives and constraints. There may also be a need for further developments of generalized system hierarchies, concepts of completeness, and analytical frameworks to facilitate hardware–software compatibility, programming flexibility, and development productivity. Furthermore, a declarative framework presupposes methods for testing and validation. For NC systems, this testing and validation would not be fully deterministic and logical, but would rather be closely interconnected with the core machine learning methodology and statistical assessment.

Table 3 summarizes the alignment of the identified research directions and concepts with the integration challenges presented in Section 1. In conclusion, there is a need for further research on interparadigmatic NC–DC communication models and virtualization to establish the transparency, reliability, and security that is typically required by large-scale distributed computing applications in cyber-physical systems and the industrial Internet. Furthermore, research on NC programming abstractions and related protocols for training, validation, and testing are required to efficiently develop, integrate, and maintain hybrid NC–DC AI solutions in such large-scale distributed digital systems of systems.

**Table 3:** Overview of research directions and concepts and their relations to neuromorphic systems integration challenges.

**Research directions and concepts**	**Neuromorphic systems integration challenges and concepts**			
	**Communication**	**Virtualization**	**Programming**	**Testing and validation**
Neuromorphic-system proxy	×	×	×	×
Neuromorphic-system simulation		⊗		⊗
Microservices	×	×		
Communication and data models	×	×		
Semantic technologies	⊗			
Embeddings	⊗			
Soft state	×	×		
PubSub messaging	×			
Graph data and querying	×	×	×	
Declarative programming			⊗	⊗
Process calculus			⊗	
Machine learning			⊗	⊗
Statistical evaluation				⊗

The symbol “⊗” indicates that a concept has been connected to neuromorphic systems in the literature discussed in this article, while “ × ” indicates that the connection is introduced here. All connections indicated in this table are subjects for further research. The relations between these concepts and conventional digital computing are described in Section 3, but are not illustrated here.

## Data availability statement

The original contributions presented in the study are included in the article/supplementary material, further inquiries can be directed to the corresponding author.

## Author contributions

MN, OS, AL, UB, and FS contributed to the initial conception of the study and to the literature study. MN wrote the first draft of the manuscript. All authors contributed to the conceptual development. All authors contributed to writing and revising the manuscript, and read and approved the submitted version.

## Footnotes

^1^ https://www.i-micronews.com/products/neuromorphic-computing-and-sensing-2021/

^2^ https://sinabs.readthedocs.io/

^3^ https://synsense-sys-int.gitlab.io/samna/

^4^ https://lava-nc.org/

^5^ https://graphql.org/
